# The role of ncRNAs in neuroblastoma: mechanisms, biomarkers and therapeutic targets

**DOI:** 10.1186/s40364-022-00368-2

**Published:** 2022-04-07

**Authors:** Shaohui Huang, Naying Gong, Jiangbin Li, Mingye Hong, Li Li, Ling Zhang, Hua Zhang

**Affiliations:** 1 Institute of Laboratory Medicine, Guangdong Provincial Key Laboratory of Medical Molecular Diagnostics, School of Medical Technology, Guangdong Medical University, Dongguan, 523808 China; 2 Health Science Center, University of Texas, Houston, 77030 USA

**Keywords:** Neuroblastoma, ncRNAs, Biomarkers, Therapeutic targets

## Abstract

Neuroblastoma (NB) is a malignant tumor in young children that originates from the neural crest of the sympathetic nervous system. Generally, NB occurs in the adrenal glands, but it can also affect the nerve tissues of the neck, chest, abdomen, and pelvis. Understanding the pathophysiology of NB and developing novel therapeutic approaches are critical. Noncoding RNAs (ncRNAs) are associated with crucial aspects of pathology, metastasis and drug resistance in NB. Here, we summarized the pretranscriptional, transcriptional and posttranscriptional regulatory mechanisms of ncRNAs involved in NB, especially focusing on regulatory pathways. Furthermore, ncRNAs with the potential to serve as biomarkers for risk stratification, drug resistance and therapeutic targets are also discussed, highlighting the clinical application of ncRNAs in NB.

## Introduction

Neuroblastoma (NB) is the most frequent extracranial solid malignant tumor in children, accounting for 6–10% of all malignancies in this age group [1–3]. NB originates from the neural ganglia of the sympathetic nervous system and occurs in adrenal glands as well as the neurological tissue of the neck, chest, abdomen, and pelvis [4]. NB has a high degree of biological heterogeneity. The majority of low-risk NB patients regress spontaneously, whereas patients with the high-risk form only have a 50% survival rate despite intensive therapy [5]. Patients are classified into low-, medium-, and high-risk groups based on histological and biochemical characteristics associated with NB, and classification schemes are continuously being developed based on new scientific data [6]. In general, patients with NB in the low-risk group achieve a high overall survival rate through observation or surgical treatment. Patients with medium-risk disease are basically treated with chemotherapy and surgery in clinical practice, which has attracted the attention of some researchers utilizing biological markers to help further reduce inappropriate treatment [4]. Patients with high-risk disease have poor survival; however, a multimodal treatment strategy comprising surgery, radiotherapy, high-dose chemotherapy, biotherapy and immunotherapy has been used to improve their survival rates [7]. Therefore, it is still crucial for researchers to help understand the pathogenesis of the disease, identify clinically significant markers and develop novel therapy strategies for patients diagnosed with NB.

Noncoding RNAs (ncRNAs) are a heterogeneous group of molecules that are basically divided into short ncRNAs (sncRNAs) and long ncRNAs (lncRNAs) based on a cut-off length of 200 bases. Among them, microRNAs (miRNAs), lncRNAs and circular RNAs (circRNAs) are the best-known classes [8, 9]. The interaction between miRNA and mRNA generally depends on complementarity with the target gene transcript sequence [10]. LncRNAs can regulate gene expression at epigenetic, transcriptional and post transcriptional levels [11]. CircRNAs can bind to proteins competitively and act as miRNA decoys or protein scaffolds [12]. Many ncRNAs are dysregulated in NB, indicating that they play important roles in disease development [13]. In this review, we primarily illustrate some of the molecular mechanisms of miRNAs, lncRNAs and circRNAs in NB; explore their roles as biomarkers; and provide some cases for targeted therapies. This information will shed new insight into NB diagnosis, risk stratification, drug targets and novel therapeutic options.

### NcRNAs involved in gene pretranscriptional regulation in NB

**Fig. 1:**
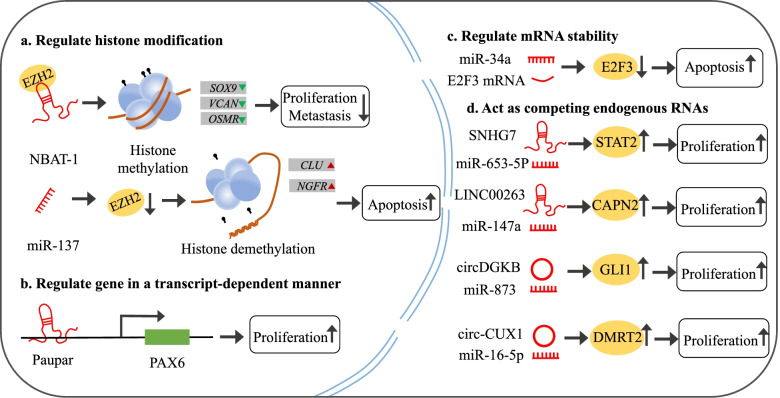
Mechanisms by which ncRNAs regulate gene expression in NB. In the nucleus, ncRNAs are involved in histone modification and transcriptional regulation. In the cytoplasm, ncRNAs generally regulate mRNA stability and act as competitive endogenous RNAs

In NB, ncRNAs are engaged in pretranscriptional gene regulation, including chromatin assembly, histone modification, and DNA methylation (Fig. 1a). The state of chromatin is determined by apparent modifiers. Chromatin rich in activated histone modifications, such as trimethylation of lysine 4 on histone H3 protein subunit (H3K4me3) and histone acetylation, is in an open state, whereas chromatin rich in inhibitory histone modifications, such as trimethylation of lysine 27 on histone H3 protein subunit (H3K27me3), is in a closed state [14, 15]. It has been reported that the upregulation of miR-137 in resveratrol-induced NB cell apoptosis leads to an imbalance in polycomb protein histone methyltransferase enhancer of zeste homolog 2 (EZH2) levels [16]. The traditional function of EZH2 as a histone methyltransferase to catalyse the modification of H3K27me3, thereby inhibiting the transcription of target genes [16, 17]. Loss of EZH2 facilitates reduction of H3K27me3 levels and activation of clusterin (CLU) and nerve growth factor receptor (NGFR), which are tumor suppressive genes in NB [16]. MiR-152 targets DNA methyltransferase 1 (DNMT1) to mediate DNA demethylation, contributing to the differentiation process induced by all-trans-retinoic acid (ATRA) and leading to the activation of Nitric oxide synthase (NOS1), which is a key gene in the differentiation of nerve cells [18]. LncRNA NBAT-1 controls the expression of target genes by interacting with EZH2. Loss of H3K27me3 from promoter regions of gene SRY-box transcription factor 9 (SOX9), versican (VCAN) and oncostatin M receptor (OSMR) leads to cell proliferation and invasion in NB [19]. The study also found that lncRNA MEG3 suppressed EZH2 expression by increasing its ubiquitination degradation. Reduced EZH2 expression inhibits the progression of NB by attenuating the H3K27me3 of many tumor suppressor genes [20]. LncRNA XIST downregulates Dickkopf-1 expression through histone methylation and promotes cell growth, invasion and migration in NB [21]. Interestingly, a lncRNA called Dali interacts with DNMT1 and causes methylation silencing of the gene POU class 3 homeobox 3 (Pou3f3), inhibiting its expression. Loss of Dali inhibits the differentiation of NB cells [22]. Knockdown of lncRNA lncUSMycN resulted in a reduction in H3K4me3 at the MYCN opposite strand gene promoter [23]. The lncRNA Paupar is suggested to be a regulator of in vivo neurogenesis. By assembling a ribonucleoprotein complex, Paupar lncRNA promotes chromatin occupancy and H3K9me3 of kinesin II associated protein 1 (KAP1) [24].

### NcRNAs involved in gene transcriptional regulation in NB

MiRNAs are well known for their regulatory role at the posttranscriptional stage. Some mature miRNAs in the nucleus, however, have been discovered to activate and silence genes at the transcriptional level [25]. Some lncRNAs can help regulate the transcriptional level of genes by influencing the transcription of adjacent coding genes and interacting with transcription factors [26].

For example, miR-373 relies on miRNA target sites in gene promoter regions to enhance the transcription of target genes [27]. In NB, miR-558 binds to the heparanase promoter through a promoter binding site to enhance its transcriptional activity and promote the transcription and protein expression of heparanase [28].

For lncRNAs, binding to proteins and transporting the complex to a specific target through direct or indirect interactions with DNA, regulating the gene expression of neighbouring or distant genes, is a key regulatory mechanism (Fig. 1b). Both locally and distantly, the lncRNA Paupar regulates paired box 6 (Pax6) in a transcription-dependent manner. Paupar enrichment around promoter binding sites can serve as a transcriptional regulator and function in trans [29]. LncRNA FOXD3-AS1 may play a role in inhibiting the poly (ADP-ribose) polymerase 1 (PARP1)-mediated CCCTC-binding factor (CTCF) transcription complex by interacting with the domain of the PARP1 protein [30]. Bioinformatics analysis shows that lncRNA SNHG16 regulates cell proliferation in NB though the transcriptional pathway [31].

### NcRNAs involved in gene posttranscriptional regulation in NB

Most miRNAs are associated with the process of gene posttranscriptional regulation. It can degrade target mRNA or inhibit its translation by pairing with target mRNA-specific bases, thus affecting the expression of target mRNA [32] (Fig. 1c). By regulating selective splicing of mRNA precursors, acting as competing endogenouse RNAs (ceRNAs), or forming complexes with proteins and changing their localization, lncRNAs can play a role in gene posttranscriptional regulation [33] (Fig. 1d). CircRNAs can also impact the expression of parental gene mRNA by interacting with RNA-binding proteins. In addition, during the formation of circRNAs, competitive complementary pairing between introns can affect mRNA expression and possibly protein translation.

miRNA-497 targets a tyrosine kinase modulator called WEE1 and promotes NB cell apoptosis [34]. By regulating the expression of matrix metalloproteinase-9 (MMP9), miR-15a facilitates NB migration [35]. The stability of paired like homeobox 2B (PHOX2B) mRNA was decreased by miRNA-204 through 3’UTR-mediated downregulation in NB cell lines [36]. In addition, research has revealed that lncRNA SNHG1 can interact with the RNA binding protein matrin-3 (MATR3), and the latter might be associated with RNA splicing to promote the progression of NB [37]. MIAT has been reported as a subnuclear lncRNA that can interfere with alternative splicing and lead to an increased risk of nervous system tumors [38]. The lncRNA MYCNOS-01 modulates MYCN expression through posttranscriptional regulation, and the growth of MYCN-amplified NB cell lines is inhibited by reducing the expression of MYCNOS-01 [39]. LncRNA NHEG1 binds to and stabilizes the DEAD-box helicase 5 (DDX5) protein, leading to activation of β-catenin and enhancing the expression of target genes related to the progression of NB [40]. Interacting with miR-653-5p to act as a ceRNA, lncRNA SNHG7 regulates signal transducer and activator of transcription 2 (STAT2) expression levels in NB cells [41]. In addition, by targeting the miR-873/ GLI family zinc finger 1 (GLI1) axis and miR-16-5p/ doublesex and mab-3 related transcription factor 2 (DMRT2) axis, circDGKB and circ-CUX1 promote the development of NB [42, 43]. CircAGO2 facilitates the transport of ELAV like RNA binding protein 1 (HuR) from the nucleus to the cytoplasm, thus restraining AGO2/miRNA-related gene silencing to promote NB occurrence and invasion [44].

### NcRNAs involved in pathway regulation in NB

**Fig. 2:**
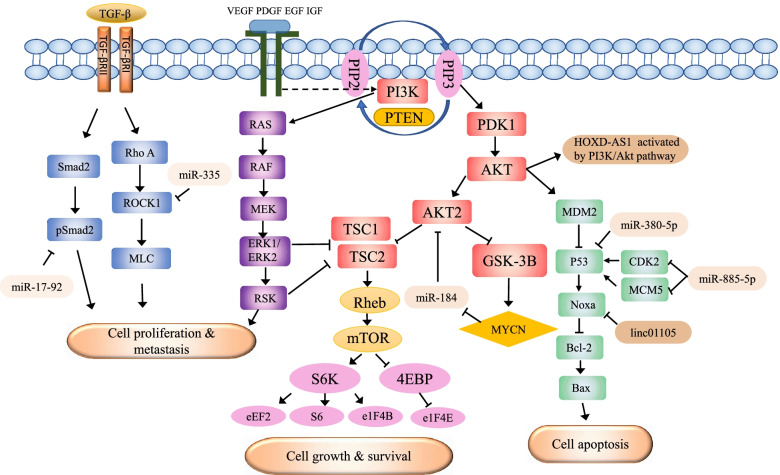
General description of the signalling pathways in which ncRNAs are involved in NB. NcRNAs participate in cell proliferation, migration, invasion and apoptosis in NB by regulating these signalling pathways

An increasing number of studies have demonstrated that the phosphoinositide 3-kinase (PI3K)/protein kinase B (AKT) pathway, P53 and transforming growth factor-β (TGF-β) signalling pathways play crucial roles in cancers, including NB [45–48]. Here, we summarized the ncRNAs currently found to participate in the development of NB by regulating these signalling pathways (Fig. 2).

#### PI3K signalling pathway

In primary NB, the PI3K/AKT pathway is often activated by serine or threonine phosphorylation and is associated with poor clinical outcomes [46]. MiRNA-184 inhibits the survival of NB cells by directly binding to the 3’UTR of AKT2 mRNA, which is the main downstream effector of the PI3K pathway [49]. MiRNA-H4-5p promotes growth, metastasis and cycle progression in a NB cell line through the PI3K/AKT pathway mediated by p16 protein [50]. The lncRNA SNHG16 may participate in the regulation of polo like kinase 4 (PLK4) expression by forming a sponge with miR-338-3p. In addition, in cisplatin-resistant NB cell lines, SNHG16 may influence the activation of the PI3K/AKT signalling pathway [51]. Activated by the PI3K/AKT signalling pathway, lncRNA HOXD-AS1 is associated with cell differentiation induced by retinoid acid [52].

#### TGF-β signalling pathway

The TGF-β pathway is regulated by the cellular environment and its integration with other signalling pathways [53]. In cancer, this pathway is associated with the cell suppression of early tumors and the proliferation, invasiveness, angiogenesis and carcinogenesis of advanced tumors [54]. It was found that miR-17-92 is an effective TGF-β signalling inhibitor in NB. The activation of miR-17-92 not only directly inhibits TGF-β responsive genes but also downregulates multiple effectors along the TGF-β pathway [47]. MiRNA-335 targets and downregulates genes associated with TGF-β noncanonical signalling, including Rho associated coiled-coil containing protein kinase 1 (ROCK1) and mitogen-activated protein kinase 1 (MAPK1), thereby reducing the phosphorylation of the motor protein myosin light chain (MLC). MLC phosphorylation subsequently causes the remarkable inhibition of invasion and migration in NB cells [55].

#### P53 signalling pathway

As a tumor suppressor protein, p53 regulates the expression of many genes involved in growth and cell cycle inhibition, apoptosis, differentiation and acceleration of DNA repair [56]. Inactivation of the transcription factor p53 through direct mutation or genetic aberration is a hallmark of almost all tumors. Interestingly, TP53 gene mutation rarely occurs in NB, suggesting that inhibitors may be involved in the regulation of p53 activity [57, 58]. MiR-380-5p represses the high levels of p53 expression, which is associated with poor prognosis in MYCN-expanded NB [59]. miR-885-5P causes the accumulation of p53 protein, thus leading to the activation of the P53 signalling pathway. Enforced expression of miR-885-5p resulted in the downregulation of cyclin-dependent kinase (CDK2) and mini-chromosome maintenance protein (MCM5), which could inhibit cell growth and induce senescence and apoptosis in NB cells [58]. To identify lncRNAs that are differentially expressed between tumor and adjacent tissues, Weitao Tang et al. found that highly expressed lncRNA HCN3, lncRNA linc01105 and downregulated lncRNA MEG3 probably play a significant role in NB through the mechanisms of p53 pathway members [48].

### NcRNAs involved in the regulation of the epithelial-to-mesenchymal transition

Epithelial-to-mesenchymal transition (EMT) is closely related to the metastatic potential of tumors, which endows cancer cells with the ability of metastasis and invasion [60]. It has been well known that several signalling pathways play important roles in the regulation of EMT, including TGFβ, Wnt and PI3K/AKT pathways [61]. By targeting doublecortin like kinase 1 (DCLK1) mRNA, miR-424 inhibits invasion and EMT in NB cells [62]. During embryogenesis, the expression of the SOX family members (SOX 8, 9, and 10) and other factors induces the loss of E-cadherins and activation of MMPs to drive the mesenchymal transformation, and Wnt signalling further drives the differentiation of neural crest cells [7]. β-Catenin actives genes that act as transcription factors in the nucleus to drive the EMT. LncRNA pancEts-1 binds to heterogeneous nuclear ribonucleoprotein K (hnRNPK) to promote interaction with β-catenin and accelerate the growth, metastasis and invasion of NB cells [63]. LncRNA MEG3 inhibits EMT through the mTOR signalling pathway, thereby inhibiting NB metastasis [64]. Another study showed that lncRNA SNHG7 and SNHG16 regulates EMT and promotes NB by interacting with miR-653-5P or miR-542-3p [41, 65].

### NcRNAs as biomarkers in NB

**Fig. 3:**
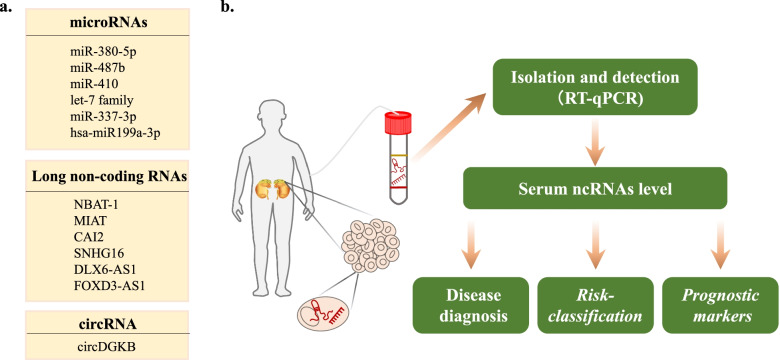
NcRNAs as tumor biomarkers and their application. **a** Some ncRNAs identified as potential biomarkers for NB. **b** Further application of ncRNA biomarkers by detecting their expression in serum

The clinical features of NB are highly heterogeneous. Major treatments for NB include surgical resection, radiotherapy and immunotherapy [66]. Despite major advancements in patient treatment, high-risk NBs are still associated with a poor prognosis with a long-term survival rate of less than 50% [67]. Pediatric oncology continues to struggle with high-risk NB. However, low-risk patients under the age of 18 months with favourable biology often regress without treatment. It is suggested that new methods for the early detection and prediction of high−/low-risk NB should be explored to improve treatment outcomes and reduce the possibility of overtreatment [68]. Some studies have revealed that ncRNAs play a significant role in assessing NB risk stratification, prognosis and drug resistance (Fig. 3, Table 1). The discovery of these markers plays a guiding role in customizing more treatment modalities.

**Table 1:** NcRNAs as risk-classification biomarkers in NB

Biomarker	Alter-ation	Study sample	Mechanism	Risk-classification	Ref
miR-29a + miR-30c + miR-95 + miR-128a + miR-128b + miR-137 + miR-138 + miR-148a + miR-195 + age + Dicer+Drosha	–	66 primary NB specimens	–	Separate patients into four distinct patterns	[69]
miR-487b + miR-410	Down	227 patients staged from INSS	–	High risk	[70]
hsa-miR199a-3p	Up	17 NB specimens	decreasing NEDD4 expression	poor prognosis	[67]
CAI2	Up	62 primary NB specimens	Regulating P16 expression	High risk	[71]
FOXD3-AS1	–	Dataset of 88 NB cases and 64 neuroblastic tumours with GEO accession	Repressing activation of CTCF	favourable outcome	[30]
NBAT-1	Down	three low-risk and 12 high-risk tumours	Activation of NRSF/REST	adverse outcome	[19]
SNHG7	Up	92 pairs of NB specimens and adjacent non-tumour specimens	interact with miR-653-5p	poor prognosis	[41]
DLX6-AS1	Up	36 NB specimens	interact with miR-107	advanced TNM stage	[72]
lncRNA-uc003opf.1	–	275 patients and 531 controls	decreasing LRFN2 expression	decreased NB risk	[73]

#### NcRNAs as risk-classification biomarkers

Plausible neural network analysis (PNN) is an intelligent self-organizing neural network system that performs characteristic selection and can be utilized to predict the prognosis of patients based on cluster analysis. Ruey-Jen Lin et al. suggested that the multivariate pattern of 15 biomarkers, including a 12-miRNA signature, can distinguish between short-term and long-term survival rates in patients with NB through PNN analysis [69]. Loss of chromosome 11q material is one of the criteria for risk stratification of NB and is often associated with poor clinical outcomes. Using miRNA expression profiling, Patrick G. Buckley et al. suggested that the 11q gene subgroup can be separated into two groups. These subgroups have significant differences in the clinical outcomes and overall frequency of segmental genomic imbalances, suggesting more subtle stratification prior to treatment [74]. In NB clinical samples, the downregulation of miR-337-3p may be connected to advanced NB stages. Patients with high miR-337-3p levels may have a survival advantage [75]. Independent of clinical covariates, miR-410 and miR-487b indicate predictive value for stratification, representing potential relapse biomarkers in favourable NB [70]. It was found that the expression of hsa-miR199a-3p, significantly upregulated in plasma exosomes of NB patients, was associated with high-risk NB [76].

In NB, lncRNA CAI2, which is located at 9p21, might contribute to abnormal p16 expression and is significantly correlated with advanced disease and poor clinical outcomes [71]. Hypermethylation of the lncRNA NBAT-1 promoter leads to low expression of NBAT-1 in high-risk NB, which can serve as a risk factor to predict NB patients with different subtypes [19]. LncRNA MIAT was found to have tumor-promoting effects in NB cell lines and has the potential to serve as a biomarker for neuroblastoma [38]. LncRNA SNHG7 is involved in the development of NB through the miR-653-5p/STAT2 pathway, and the upregulated SNHG7 is associated with poor survival of NB patients [41]. Yes1 associated transcriptional regulator (YAP1) expression is regulated by the lncRNA DLX6-AS1, which interacts with miR-497-5p and is positively associated with advanced-stage NB [72]. LncRNA FOXD3-AS1 is a good prognostic marker for patients with NB through the mining of public microarray datasets [30]. Several lncRNA polymorphisms have been discovered to predict the risk of NB, including lncRNA-uc003opf.1, which may be related to low NB risk [73].

Although circRNAs have not been shown to be risk-classification markers for NB, circDGKB promotes NB development by targeting the miR-873/GLI1 axis, indicating that it could be utilized as a new diagnostic marker for NB [42].

#### NcRNAs as drug resistance biomarkers

Apoptosis, cell growth-associated pathways and DNA damage repair have all been identified as mechanisms for acquired multidrug resistance in cancer cells [77]. MYCN transcription factors influence drug resistance in NB by modulating a number of genes, including those involved in drug outflow. Furthermore, the deletion of chromosome 11q is also related to the development of resistance to multiple drugs. Multidrug resistance is one of the most difficult challenges to overcome in the treatment of NB. Several studies have suggested that miRNA imbalances are also involved in drug resistance in NB, which enriches the mechanistic network of multidrug resistance in cancer [78–81]. miR-155 and miR-21 contribute to cisplatin drug resistance in a NB xenograft mouse model [78]. Exosmic miR-155 directly targets telomeric repeat binding factor 1 (TERF1), a telomerase inhibitor that impacts telomerase activity, which is associated with drug resistance and poor prognosis in a variety of malignant cancers. In another study, by targeting miR-520f, neuronal apoptosis inhibitor protein produced drug resistance through apoptosis inhibition in non-MYCN amplified tumor cells [79]. In a NB model cell line, B. Marengo et al. demonstrated that etoposide resistance is linked to miRNA-15a/16–1 downregulation [81].

MiRNAs are not only related to drug resistance but also connected to chemotherapeutic effects. miR-340 inhibits SOX2 transcription factors directly by targeting their 3′-UTR [82]. This finding explains the mechanism by which ATRA downregulates SOX2, whereas the latter helps stem cell undifferentiation [83, 84]. By enhancing apoptosis, miR-204 remarkably improved the susceptibility of NB cell lines to cisplatin [85]. Treatment with cisplatin led to a significant increase in apoptosis of MYCN-amplified tumor cells, indicating a synergistic effect between cisplatin and miR-497 and therefore suggesting a potential neuroblastoma treatment [34].

However, most studies indicating miRNAs as NB biomarkers had major limitations. For example, the drug-resistant NB cell models were based on in vitro selection, or the studies had limited clinical cohort sizes and patient numbers. Indeed, a larger patient cohort considering patients with various known drug resistance features might be used to assess the therapeutic utility of the hypothesized miRNA drug resistance biomarkers.

In recent years, several studies have reported lncRNAs as drug resistance markers of NB. Low expression of the lncRNA NBAT-1 generates NB resistance to genotoxic drugs by regulating the nuclear p53 pathway [86]. Through the miR-144-3p/ histone deacetylase 8 (HDAC8) axis, the lncRNA NORAD increases NB growth and adriamycin resistance [87]. In NB, silencing the lncRNA SNHG16 gene can help diminish cisplatin resistance and improve the antitumor activity of cisplatin [51]. Deletion of lncRNA SNHG7 enhances cisplatin sensitivity by modulating the miR-329-3p/myosin X axis [88].

To date, circRNAs have not been reported as drug resistance markers of NB. However, circRNAs play a significant role in drug resistance in a variety of different malignancies. For example, circASAP1 overexpression enhances glioblastoma cell proliferation and resistance to decontazolamide [89]. Through autophagy activation controlled by miR-142-3p/rock2, circCUL2 might regulate the cisplatin sensitivity of gastric cancer [90]. CircUHRF1 might contribute to the resistance of anti-PD1 immunotherapy by hepatocellular carcinoma cells, revealing a potential treatment strategy for patients with hepatocellular carcinoma [91].

## NcRNAs as therapeutic targets in NB

General problems faced in NB treatment include poor therapy response and even resistance to classical chemotherapeutics. Furthermore, the therapeutic options for advanced NB are limited, and severe adverse effects are frequently reported. Deeper research on ncRNA mechanisms in NB will contribute to the recognition of new targets for therapeutic intervention. Furthermore, treatment methods directly targeting or utilizing ncRNA molecules may help to improve NB treatment.

It is worth mentioning that MYCN is amplified in approximately 50% of high-risk NB and is closely associated with poor prognosis [92, 93]. Studies showed that amplification of MYCN promotes the development and progression of high-risk NB [5, 94]. Due to the complicated structure of MYCN, it is difficult to be a direct therapeutic target [95]. However, a number of miRNAs have been found to directly target MYCN and inhibit NB cell proliferation, such as miRNA let-7e, miR-202, miR-375 and miR-204 [96–98], suggesting that overexpression of these miRNAs may provide a novel therapeutic strategy for MYCN-amplified NB.

**Fig. 4:**
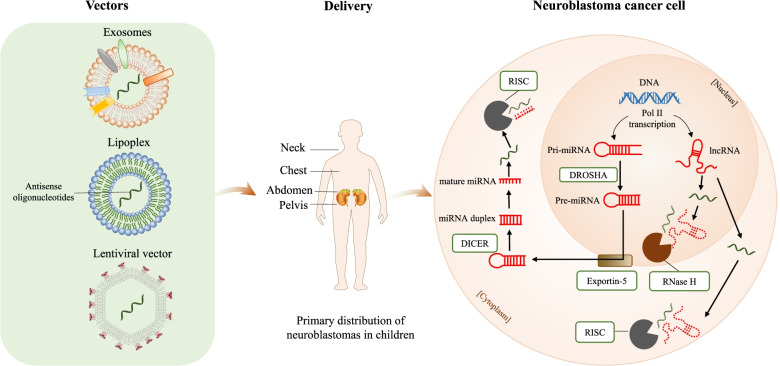
Principle of single-stranded oligonucleotides targeting miRNAs and lncRNAs. Single-stranded antisense oligonucleotides can induce miRNA degradation through the recognition and processing of the RNA-induced silencing complex (RISC). RNaseH and RISC can recognize and induce the degradation of lncRNAs. Several types of vectors allow oligonucleotides to penetrate into the cytoplasm; thus, a variety of drug delivery strategies, including lipoplexes, polymeric nanoparticles, exosomes, and expression vectors, can be used

RNA-based therapies, including RNAi technology, antisense oligonucleotides (ASO), and small molecule inhibitors have been developed [99–101]. The drug delivery strategies include liposomes, polymeric nanoparticles, exosomes and expression vectors and so on (Fig. 4) Among them, nanoparticle-based treatments have generated increasing research interest as a promising alternative to traditional cancer treatment methods [102, 103]. Safe and effective nanocarriers that transport miR-34a and let-7b to NB cells can considerably reduce tumor growth in pseudometastatic models, cause cell death, and enhance the survival rate of mice in pseudometastatic models [104]. According to the study by Mohammadniaei M. et al., a multifunctional nanobiohybrid material composed of a newly developed RNA three-way junction structure in which one leg inhibits miRNA-17 and the other leg releases retinoic acid could completely differentiate SH-SY5Y NB cells into neurons [105]. Engineered pH-sensitive and stable nanovesicles protect miRNAs from RNases. When injected intravenously, these nanovesicles can be delivered to NB xenograft tumors, which can effectively deliver therapeutically active miRNAs and other small RNAs [106]. Delivery of natural killer cell (NK) -derived exosomes or nanoparticles to recover miR-186 levels in NB can resume the cell-killing abilities of NK cells and promote survival, revealing its potential therapeutic effects in NB [107]. As a receptor for different molecules, such as hormones and growth factors, in a mouse orthotopic xenograft model, nanoparticles containing miR-34a coupled to a disialoganglioside GD2 antibody were shown to improve tumor-specific delivery, resulting in a substantial reduction in tumor growth, increased apoptosis, and reduced angiogenesis [108].

In addition, conditional replication of oncolytic viruses is an attractive choice for the treatment of cancers because viruses can replicate in proliferating tumor cells and destroy them, and they have the ability to induce an immune response to confront tumor cells. Oncolytic viruses show bright prospects for high-risk NB treatment. Ramachandran M et al. constructed SFVmiRT by inserting the target sequences of miR124, miR125, and miR134 into the semliki Forest virus (SFV)-4 genome. SFVmiRT not only reduces the neurovirulence of SFV4 in mice but also maintains its replication and oncolytic ability in NB [109].

## Conclusion and discussion

With the development of sequencing technology, an increasing number of ncRNAs have been discovered to participate in the gene regulation network of tumorigenesis, which has caused a significant change in our understanding of tumor formation and treatment. In NB, ncRNAs have been found to play a critical function in epigenetic and genetic regulation [25, 51]. In addition, ncRNAs have been shown to be related to NB-related clinicopathological parameters, such as survival, prognosis and recurrence. Although circRNAs are related to various processes in a variety of cancers, there are relatively few studies on circRNAs in NB. Notably, the role of ncRNA in tumor immunity can not be underestimated. Studies have shown that the recognition and clearance of tumor cells by immune cells also depend on the regulation of ncRNAs [110], which deserves further research.

The ideal biomarker should have the characteristics of good stability, strong specificity and high sensitivity. NcRNAs either have significant tissue specificity or can be detected in serum or other body fluids. These characteristics indicate that ncRNAs are suitable biomarkers for NB. In particular, the detection of ncRNAs in serum is particularly promising due to the advantages of a noninvasive and convenient procedure. In addition, RNA molecules contained in exosomes, apoptotic bodies, and also have better research prospects given that these molecules are shielded from RNase in fluids. To date, ncRNA-based therapy has not been assessed in clinical trials in NB. However, many promising methods to regulate miRNAs at a therapeutic level are in the preclinical stage. Research on the utility of lncRNAs and circRNAs in NB therapy are lacking.

In summary, ncRNA studies have enriched the understanding of NB. However, information on the role and mechanism of ncRNAs in NB is only starting to appear. The application of ncRNAs in cancer treatment is a promising field. However, it faces many challenges, including reduction of toxicity, enhancement of efficient and accurate delivery. Further ncRNA-focused research and clinical practice will provide novel biomarkers and new treatment strategies for the clinical application of ncRNAs in NB.

## Data Availability

Not applicable for this article.

## Abbreviations

NB: Neuroblastoma
ncRNA: Noncoding RNA
sncRNA: Short noncoding RNA
lncRNA: Long noncoding RNA
miRNAs: microRNA
circRNA: circular RNA
H3K4me3: Trimethylation of lysine 4 on histone H3 protein subunit
H3K27me3: Trimethylation of lysine 27 on histone H3 protein subunit
EZH2: Polycomb protein histone methyltransferase enhancer of zeste homolog 2
CLU: Clusterin
NGFR: Nerve growth factor receptor
DNMT1: DNA methyltransferase 1
ATRA: All-trans-retinoic acid
NOS1: Nitric oxide synthase
SOX: SRY-box transcription factor
VCAN: Versican
OSMR: Oncostatin M receptor
Pou3f3: POU class 3 homeobox 3
KAP1: Kinesin II associated protein 1
Pax6: Paired box 6
PARP1: Poly (ADP-ribose) polymerase 1
CTCF: CCCTC-binding factor
ceRNA: Competing endogenouse RNA
MMP: Matrix metalloproteinase
PHOX2B: Paired like homeobox 2B
MATR3: Matrin-3
DDX5: DEAD-box helicase 5
STAT2: Transducer and activator of transcription 2
GLI1: GLI family zinc finger 1
DMRT2: Doublesex and mab-3 related transcription factor 2
HuR: ELAV like RNA binding protein 1
PI3K: Phosphoinositide 3-kinase
AKT: Protein kinase B
TGF-β: Transforming growth factor-β
PLK4: Polo like kinase 4
ROCK: Rho associated coiled-coil containing protein kinase
MAP K1: Mitogen-activated protein kinase 1
MLC: Myosin light chain
CDK2: Cyclin-dependent kinase
MCM5: Mini-chromosome maintenance protein
DCLK1: Doublecortin like kinase 1
EMT: Epithelial-to-mesenchymal transition
hnRNPK: Heterogeneous nuclear ribonucleoprotein K
PNN: Plausible neural network analysis
TERF1: Telomeric repeat binding factor 1
HDAC8: Histone deacetylase 8
ASO: Antisense oligonucleotide
NK: Natural killer cell
SFV: Semliki Forest virus

## References

[1] Brodeur GM (2003). Neuroblastoma: biological insights into a clinical enigma. Nat Rev Cancer.

[2] Song H, Li D, Wang X, Fang E, Yang F, Hu A (2020). HNF4A-AS1/hnRNPU/CTCF axis as a therapeutic target for aerobic glycolysis and neuroblastoma progression. J Hematol Oncol.

[3] Tumino N, Weber G, Besi F, Del Bufalo F, Bertaina V, Paci P (2021). Polymorphonuclear myeloid-derived suppressor cells impair the anti-tumor efficacy of GD2.CAR T-cells in patients with neuroblastoma. J Hematol Oncol.

[4] Tsubota S, Kadomatsu K (2018). Origin and initiation mechanisms of neuroblastoma. Cell Tissue Res.

[5] Maris JM (2010). Recent advances in neuroblastoma. N Engl J Med.

[6] Ackermann S, Cartolano M, Hero B, Welte A, Kahlert Y, Roderwieser A (2018). A mechanistic classification of clinical phenotypes in neuroblastoma. Science..

[7] Louis CU, Shohet JM (2015). Neuroblastoma: molecular pathogenesis and therapy. Annu Rev Med.

[8] Decruyenaere P, Offner F, Vandesompele J (2021). Circulating RNA biomarkers in diffuse large B-cell lymphoma: a systematic review. Exp Hematol Oncol..

[9] Huang X, Huang L, Xie Q, Zhang L, Huang S, Hong M (2021). LncRNAs serve as novel biomarkers for diagnosis and prognosis of childhood ALL. Biomark Res..

[10] Sun YM, Chen YQ (2020). Principles and innovative technologies for decrypting noncoding RNAs: from discovery and functional prediction to clinical application. J Hematol Oncol.

[11] Sanchez Calle A, Kawamura Y, Yamamoto Y, Takeshita F, Ochiya T (2018). Emerging roles of long non-coding RNA in cancer. Cancer Sci.

[12] Shen H, Liu B, Xu J, Zhang B, Wang Y, Shi L (2021). Circular RNAs: characteristics, biogenesis, mechanisms and functions in liver cancer. J Hematol Oncol.

[13] Kim SH, Lim KH, Yang S, Joo JY (2021). Long non-coding RNAs in brain tumors: roles and potential as therapeutic targets. J Hematol Oncol.

[14] Voigt P, Tee WW, Reinberg D. A double take on bivalent promoters. Genes Dev. 2013;27(12):1318–38. 10.1101/gad.219626.113 PMC3701188 23788621

[15] Shen H, Lan Y, Zhao Y, Shi Y, Jin J, Xie W (2020). The emerging roles of N6-methyladenosine RNA methylation in human cancers. Biomark Res..

[16] Ren X, Bai X, Zhang X, Li Z, Tang L, Zhao X (2015). Quantitative nuclear proteomics identifies that miR-137-mediated EZH2 reduction regulates resveratrol-induced apoptosis of neuroblastoma cells. Mol Cell Proteomics.

[17] Duan R, Du W, Guo W (2020). EZH2: a novel target for cancer treatment. J Hematol Oncol.

[18] Das S, Foley N, Bryan K, Watters KM, Bray I, Murphy DM (2010). MicroRNA mediates DNA demethylation events triggered by retinoic acid during neuroblastoma cell differentiation. Cancer Res.

[19] Pandey GK, Mitra S, Subhash S, Hertwig F, Kanduri M, Mishra K (2014). The risk-associated long noncoding RNA NBAT-1 controls neuroblastoma progression by regulating cell proliferation and neuronal differentiation. Cancer Cell.

[20] Zhuo ZJ, Zhang R, Zhang J, Zhu J, Yang T, Zou Y, et al. Associations between lncRNA MEG3 polymorphisms and neuroblastoma risk in Chinese children. Aging*.* 2018;10(3):481–91. 10.18632/aging.101406 PMC5892699 29615542

[21] Zhang J, Li WY, Yang Y, Yan LZ, Zhang SY, He J (2019). LncRNA XIST facilitates cell growth, migration and invasion via modulating H3 histone methylation of DKK1 in neuroblastoma. Cell Cycle.

[22] Chalei V, Sansom SN, Kong L, Lee S, Montiel JF, Vance KW (2014). The long non-coding RNA Dali is an epigenetic regulator of neural differentiation. Elife..

[23] Liu PY, Atmadibrata B, Mondal S, Tee AE, Liu T (2016). NCYM is upregulated by lncUSMycN and modulates N-Myc expression. Int J Oncol.

[24] Pavlaki I, Alammari F, Sun B, Clark N, Sirey T, Lee S (2018). The long non-coding RNA promotes KAP1-dependent chromatin changes and regulates olfactory bulb neurogenesis. EMBO J.

[25] Fan L, Lai R, Ma N, Dong Y, Li Y, Wu Q (2021). miR-552-3p modulates transcriptional activities of FXR and LXR to ameliorate hepatic glycolipid metabolism disorder. J Hepatol.

[26] Statello L, Guo CJ, Chen LL, Huarte M (2021). Gene regulation by long non-coding RNAs and its biological functions. Nat Rev Mol Cell Biol..

[27] Place RF, Li LC, Pookot D, Noonan EJ, Dahiya R (2008). MicroRNA-373 induces expression of genes with complementary promoter sequences. Proc Natl Acad Sci U S A.

[28] Qu H, Zheng L, Pu J, Mei H, Xiang X, Zhao X (2015). miRNA-558 promotes tumorigenesis and aggressiveness of neuroblastoma cells through activating the transcription of heparanase. Hum Mol Genet.

[29] Vance KW, Sansom SN, Lee S, Chalei V, Kong L, Cooper SE (2014). The long non-coding RNA Paupar regulates the expression of both local and distal genes. EMBO J.

[30] Zhao X, Li D, Huang D, Song H, Mei H, Fang E (2018). Risk-Associated Long Noncoding RNA FOXD3-AS1 Inhibits Neuroblastoma Progression by Repressing PARP1-Mediated Activation of CTCF. Mol Ther.

[31] Yu Y, Chen F, Yang Y, Jin Y, Shi J, Han S (2019). lncRNA SNHG16 is associated with proliferation and poor prognosis of pediatric neuroblastoma. Int J Oncol.

[32] Welch C, Chen Y, Stallings RL. MicroRNA-34a functions as a potential tumor suppressor by inducing apoptosis in neuroblastoma cells. Oncogene. 2007;26(34):5017–22. 10.1038/sj.onc.1210293 17297439

[33] Lee WJ, Shin CH, Ji H, et al. hnRNPK-regulated LINC00263 promotes malignant phenotypes through miR-147a/CAPN2. Cell Death Dis. 2021;12(4):290. 10.1038/s41419-021-03575-1 PMC7969774 33731671

[34] Creevey L, Ryan J, Harvey H, Bray IM, Meehan M, Khan AR (2013). MicroRNA-497 increases apoptosis in MYCN amplified neuroblastoma cells by targeting the key cell cycle regulator WEE1. Mol Cancer.

[35] Xin C, Buhe B, Hongting L, Chuanmin Y, Xiwei H, Hong Z (2013). MicroRNA-15a promotes neuroblastoma migration by targeting reversion-inducing cysteine-rich protein with Kazal motifs (RECK) and regulating matrix metalloproteinase-9 expression. FEBS J.

[36] Bachetti T, Di Zanni E, Ravazzolo R, Ceccherini I (2015). miR-204 mediates post-transcriptional down-regulation of PHOX2B gene expression in neuroblastoma cells. Biochim Biophys Acta.

[37] Yang TW, Sahu D, Chang YW, Hsu CL, Hsieh CH, Huang HC (2019). RNA-Binding Proteomics Reveals MATR3 Interacting with lncRNA SNHG1 To Enhance Neuroblastoma Progression. J Proteome Res.

[38] Bountali A, Tonge DP, Mourtada-Maarabouni M (2019). RNA sequencing reveals a key role for the long non-coding RNA MIAT in regulating neuroblastoma and glioblastoma cell fate. Int J Biol Macromol.

[39] O'Brien EM, Selfe JL, Martins AS, Walters ZS, Shipley JM (2018). The long non-coding RNA MYCNOS-01 regulates MYCN protein levels and affects growth of MYCN-amplified rhabdomyosarcoma and neuroblastoma cells. BMC Cancer.

[40] Zhao X, Li D, Yang F, Lian H, Wang J, Wang X (2020). Long Noncoding RNA NHEG1 Drives β-Catenin Transactivation and Neuroblastoma Progression through Interacting with DDX5. Mol Ther.

[41] Chi R, Chen X, Liu M, Zhang H, Li F, Fan X (2019). Role of SNHG7-miR-653-5p-STAT2 feedback loop in regulating neuroblastoma progression. J Cell Physiol.

[42] Yang J, Yu L, Yan J, Xiao Y, Li W, Xiao J (2020). Circular RNA DGKB Promotes the Progression of Neuroblastoma by Targeting miR-873/GLI1 Axis. Front Oncol.

[43] Zhang X, Zhang J, Liu Q, Zhao Y, Zhang W, Yang H (2020). Circ-CUX1 Accelerates the Progression of Neuroblastoma via miR-16-5p/DMRT2 Axis. Neurochem Res.

[44] Chen Y, Yang F, Fang E, Xiao W, Mei H, Li H (2019). Circular RNA circAGO2 drives cancer progression through facilitating HuR-repressed functions of AGO2-miRNA complexes. Cell Death Differ.

[45] Wu J, Cang S, Liu C, Ochiai W, Chiao JW (2020). Development of human prostate cancer stem cells involves epigenomic alteration and PI3K/AKT pathway activation. Exp Hematol Oncol..

[46] Opel D, Poremba C, Simon T, Debatin K-M, Fulda S (2007). Activation of Akt predicts poor outcome in neuroblastoma. Cancer Res.

[47] Mestdagh P, Boström A-K, Impens F, Fredlund E, Van Peer G, De Antonellis P (2010). The miR-17-92 microRNA cluster regulates multiple components of the TGF-β pathway in neuroblastoma. Mol Cell.

[48] Tang W, Dong K, Li K, Dong R, Zheng S (2016). MEG3, HCN3 and linc01105 influence the proliferation and apoptosis of neuroblastoma cells via the HIF-1alpha and p53 pathways. Sci Rep.

[49] Foley NH, Bray IM, Tivnan A, Bryan K, Murphy DM, Buckley PG (2010). MicroRNA-184 inhibits neuroblastoma cell survival through targeting the serine/threonine kinase AKT2. Mol Cancer.

[50] Zhao H, Zhang C, Hou G, Song J (2015). MicroRNA-H4-5p encoded by HSV-1 latency-associated transcript promotes cell proliferation, invasion and cell cycle progression via p16-mediated PI3K-Akt signaling pathway in SHSY5Y cells. Int J Clin Exp Med.

[51] Xu Z, Sun Y, Wang D, Sun H, Liu X (2020). SNHG16 promotes tumorigenesis and cisplatin resistance by regulating miR-338-3p/PLK4 pathway in neuroblastoma cells. Cancer Cell Int.

[52] Yarmishyn AA, Batagov AO, Tan JZ, Sundaram GM, Sampath P, Kuznetsov VA (2014). HOXD-AS1 is a novel lncRNA encoded in HOXD cluster and a marker of neuroblastoma progression revealed via integrative analysis of noncoding transcriptome. BMC Genomics.

[53] Derynck R, Turley SJ, Akhurst RJ (2021). TGFβ biology in cancer progression and immunotherapy. Nat Rev Clin Oncol.

[54] Massagué J (2008). TGFbeta in Cancer. Cell..

[55] Lynch J, Fay J, Meehan M, Bryan K, Watters KM, Murphy DM (2012). MiRNA-335 suppresses neuroblastoma cell invasiveness by direct targeting of multiple genes from the non-canonical TGF-beta signalling pathway. Carcinogenesis..

[56] Lacroix M, Riscal R, Arena G, Linares LK, Le Cam L (2020). Metabolic functions of the tumor suppressor p53: Implications in normal physiology, metabolic disorders, and cancer. Mol Metab.

[57] Van Maerken T, Vandesompele J, Rihani A, De Paepe A, Speleman F (2009). Escape from p53-mediated tumor surveillance in neuroblastoma: switching off the p14(ARF)-MDM2-p53 axis. Cell Death Differ.

[58] Afanasyeva EA, Mestdagh P, Kumps C, Vandesompele J, Ehemann V, Theissen J (2011). MicroRNA miR-885-5p targets CDK2 and MCM5, activates p53 and inhibits proliferation and survival. Cell Death Differ.

[59] Swarbrick A, Woods SL, Shaw A, Balakrishnan A, Phua Y, Nguyen A (2010). miR-380-5p represses p53 to control cellular survival and is associated with poor outcome in MYCN-amplified neuroblastoma. Nat Med.

[60] Dong B, Li S, Zhu S, Yi M, Luo S, Wu K (2021). MiRNA-mediated EMT and CSCs in cancer chemoresistance. Exp Hematol Oncol.

[61] Dongre A, Weinberg RA (2019). New insights into the mechanisms of epithelial-mesenchymal transition and implications for cancer. Nat Rev Mol Cell Biol.

[62] Wan MF, Yang N, Qu NY, Pan YY, Shan YQ, Li P (2020). MiR-424 suppressed viability and invasion by targeting to the DCLK1 in neuroblastoma. Eur Rev Med Pharmacol Sci.

[63] Li D, Wang X, Mei H, Fang E, Ye L, Song H (2018). Long Noncoding RNA pancEts-1 Promotes Neuroblastoma Progression through hnRNPK-Mediated β-Catenin Stabilization. Cancer Res.

[64] Ye M, Lu H, Tang W, Jing T, Chen S, Wei M (2020). Downregulation of MEG3 promotes neuroblastoma development through FOXO1-mediated autophagy and mTOR-mediated epithelial-mesenchymal transition. Int J Biol Sci.

[65] Deng D, Yang S, Wang X (2020). Long non-coding RNA SNHG16 regulates cell behaviors through miR-542-3p/HNF4α axis via RAS/RAF/MEK/ERK signaling pathway in pediatric neuroblastoma cells. Biosci Rep.

[66] Zafar A, Wang W, Liu G, Wang X, Xian W, McKeon F (2021). Molecular targeting therapies for neuroblastoma: Progress and challenges. Med Res Rev.

[67] Pinto NR, Applebaum MA, Volchenboum SL, Matthay KK, London WB, Ambros PF (2015). Advances in Risk Classification and Treatment Strategies for Neuroblastoma. J Clin Oncol.

[68] Matthay KK, Maris JM, Schleiermacher G, Nakagawara A, Mackall CL, Diller L (2016). Neuroblastoma. Nat Rev Dis Primers.

[69] Lin RJ, Lin YC, Chen J, Kuo HH, Chen YY, Diccianni MB (2010). microRNA signature and expression of Dicer and Drosha can predict prognosis and delineate risk groups in neuroblastoma. Cancer Res.

[70] Gattolliat CH, Thomas L, Ciafre SA, Meurice G, Le Teuff G, Job B (2011). Expression of miR-487b and miR-410 encoded by 14q32.31 locus is a prognostic marker in neuroblastoma. Br J Cancer.

[71] Barnhill LM, Williams RT, Cohen O, Kim Y, Batova A, Mielke JA (2014). High expression of CAI2, a 9p21-embedded long noncoding RNA, contributes to advanced-stage neuroblastoma. Cancer Res.

[72] Zhang HY, Xing MQ, Guo J, Zhao JC, Chen X, Jiang Z (2019). Long noncoding RNA DLX6-AS1 promotes neuroblastoma progression by regulating miR-107/BDNF pathway. Cancer Cell Int.

[73] Pan J, Lin H, Yang T, Yang J, Hu C, Zhu J (2020). rs11752942 A>G polymorphism decreases neuroblastoma risk in Chinese children. Cell Cycle.

[74] Buckley PG, Alcock L, Bryan K, Bray I, Schulte JH, Schramm A (2010). Chromosomal and microRNA expression patterns reveal biologically distinct subgroups of 11q- neuroblastoma. Clin Cancer Res.

[75] Xiang X, Mei H, Zhao X, et al. miRNA-337-3p suppresses neuroblastoma progression by repressing the transcription of matrix metalloproteinase 14. Oncotarget. 2015;6(26):22452–66. 10.18632/oncotarget.4311 PMC4673175 26084291

[76] Ma J, Xu M, Yin M, Hong J, Chen H, Gao Y (2019). Exosomal hsa-miR199a-3p Promotes Proliferation and Migration in Neuroblastoma. Front Oncol.

[77] Shen DW, Pouliot LM, Hall MD, Gottesman MM (2012). Cisplatin resistance: a cellular self-defense mechanism resulting from multiple epigenetic and genetic changes. Pharmacol Rev.

[78] Challagundla KB, Wise PM, Neviani P, Chava H, Murtadha M, Xu T (2015). Exosome-mediated transfer of microRNAs within the tumor microenvironment and neuroblastoma resistance to chemotherapy. J Natl Cancer Inst.

[79] Harvey H, Piskareva O, Creevey L, Alcock LC, Buckley PG, O'Sullivan MJ (2015). Modulation of chemotherapeutic drug resistance in neuroblastoma SK-N-AS cells by the neural apoptosis inhibitory protein and miR-520f. Int J Cancer.

[80] Roth SA, Knutsen E, Fiskaa T, Utnes P, Bhavsar S, Hald ØH (2016). Next generation sequencing of microRNAs from isogenic neuroblastoma cell lines isolated before and after treatment. Cancer Lett.

[81] Marengo B, Monti P, Miele M, Menichini P, Ottaggio L, Foggetti G (2018). Etoposide-resistance in a neuroblastoma model cell line is associated with 13q14.3 mono-allelic deletion and miRNA-15a/16-1 down-regulation. Sci Rep.

[82] Das S, Bryan K, Buckley PG, Piskareva O, Bray IM, Foley N (2013). Modulation of neuroblastoma disease pathogenesis by an extensive network of epigenetically regulated microRNAs. Oncogene..

[83] Gangemi RM, Griffero F, Marubbi D, Perera M, Capra MC, Malatesta P (2009). SOX2 silencing in glioblastoma tumor-initiating cells causes stop of proliferation and loss of tumorigenicity. Stem Cells.

[84] Stevanovic M (2003). Modulation of SOX2 and SOX3 gene expression during differentiation of human neuronal precursor cell line NTERA2. Mol Biol Rep.

[85] Ryan J, Tivnan A, Fay J, Bryan K, Meehan M, Creevey L (2012). MicroRNA-204 increases sensitivity of neuroblastoma cells to cisplatin and is associated with a favourable clinical outcome. Br J Cancer.

[86] Mitra S, Muralidharan SV, Di Marco M, Juvvuna PK, Kosalai ST, Reischl S (2021). Subcellular Distribution of p53 by the p53-Responsive lncRNA NBAT1 Determines Chemotherapeutic Response in Neuroblastoma. Cancer Res.

[87] Wang B, Xu L, Zhang J, Cheng X, Xu Q, Wang J (2020). LncRNA NORAD accelerates the progression and doxorubicin resistance of neuroblastoma through up-regulating HDAC8 via sponging miR-144-3p. Biomed Pharmacother.

[88] Wang SY, Wang X, Zhang CY (2020). LncRNA SNHG7 enhances chemoresistance in neuroblastoma through cisplatin-induced autophagy by regulating miR-329-3p/MYO10 axis. Eur Rev Med Pharmacol Sci.

[89] Wei Y, Lu C, Zhou P, Zhao L, Lyu X, Yin J (2021). EIF4A3-induced circular RNA ASAP1 promotes tumorigenesis and temozolomide resistance of glioblastoma via NRAS/MEK1/ERK1-2 signaling. Neuro-Oncology.

[90] Peng L, Sang H, Wei S, Li Y, Jin D, Zhu X (2020). circCUL2 regulates gastric cancer malignant transformation and cisplatin resistance by modulating autophagy activation via miR-142-3p/ROCK2. Mol Cancer.

[91] Zhang PF, Gao C, Huang XY, Lu JC, Guo XJ, Shi GM (2020). Cancer cell-derived exosomal circUHRF1 induces natural killer cell exhaustion and may cause resistance to anti-PD1 therapy in hepatocellular carcinoma. Mol Cancer.

[92] Brodeur GM, Seeger RC, Schwab M, Varmus HE, Bishop JM (1984). Amplification of N-myc in untreated human neuroblastomas correlates with advanced disease stage. Science..

[93] Seeger RC, Brodeur GM, Sather H, Dalton A, Siegel SE, Wong KY (1985). Association of multiple copies of the N-myc oncogene with rapid progression of neuroblastomas. N Engl J Med.

[94] Rickman DS, Schulte JH, Eilers M (2018). The Expanding World of N-MYC-Driven Tumors. Cancer Discov.

[95] Huang M, Weiss WA (2013). Neuroblastoma and MYCN. Cold Spring Harb Perspect Med.

[96] Buechner J, Tømte E, Haug BH, Henriksen JR, Løkke C, Flægstad T (2011). Tumour-suppressor microRNAs let-7 and mir-101 target the proto-oncogene MYCN and inhibit cell proliferation in MYCN-amplified neuroblastoma. Br J Cancer.

[97] Zhang H, Liu T, Yi S, Gu L, Zhou M (2015). Targeting MYCN IRES in MYCN-amplified neuroblastoma with miR-375 inhibits tumor growth and sensitizes tumor cells to radiation. Mol Oncol.

[98] Ooi CY, Carter DR, Liu B, Mayoh C, Beckers A, Lalwani A (2018). Network Modeling of microRNA-mRNA Interactions in Neuroblastoma Tumorigenesis Identifies miR-204 as a Direct Inhibitor of MYCN. Cancer Res.

[99] Ling H, Fabbri M, Calin GA (2013). MicroRNAs and other non-coding RNAs as targets for anticancer drug development. Nat Rev Drug Discov.

[100] Tripathi A, Kashyap A, Tripathi G, Yadav J, Bibban R, Aggarwal N (2021). Tumor reversion: a dream or a reality. Biomark Res..

[101] Harder A (2021). MEK inhibitors-novel targeted therapies of neurofibromatosis associated benign and malignant lesions. Biomark Res..

[102] Yin H, Kanasty RL, Eltoukhy AA, Vegas AJ, Dorkin JR, Anderson DG (2014). Non-viral vectors for gene-based therapy. Nat Rev Genet.

[103] Dou L, Meng X, Yang H, Dong H (2021). Advances in technology and applications of nanoimmunotherapy for cancer. Biomark Res.

[104] Di Paolo D, Pastorino F, Brignole C, Corrias MV, Emionite L, Cilli M (2020). Combined Replenishment of miR-34a and let-7b by Targeted Nanoparticles Inhibits Tumor Growth in Neuroblastoma Preclinical Models. Small..

[105] Mohammadniaei M, Yoon J, Choi HK, Placide V, Bharate BG, Lee T (2019). Multifunctional Nanobiohybrid Material Composed of Ag@Bi_2_Se_3_/RNA Three-Way Junction/miRNA/Retinoic Acid for Neuroblastoma Differentiation. ACS Appl Mater Interfaces.

[106] Boloix A, Feiner-Gracia N, Köber M, Repetto J, Pascarella R, Soriano A (2022). Engineering pH-Sensitive Stable Nanovesicles for Delivery of MicroRNA Therapeutics. Small..

[107] Schmittgen TD (2019). Exosomal miRNA Cargo as Mediator of Immune Escape Mechanisms in Neuroblastoma. Cancer Res.

[108] Tivnan A, Orr WS, Gubala V, Nooney R, Williams DE, McDonagh C (2012). Inhibition of neuroblastoma tumor growth by targeted delivery of microRNA-34a using anti-disialoganglioside GD2 coated nanoparticles. PLoS One.

[109] Ramachandran M, Yu D, Dyczynski M, Baskaran S, Zhang L, Lulla A (2017). Safe and Effective Treatment of Experimental Neuroblastoma and Glioblastoma Using Systemically Delivered Triple MicroRNA-Detargeted Oncolytic Semliki Forest Virus. Clin Cancer Res.

[110] Zhang L, Xu X, Su X (2020). Noncoding RNAs in cancer immunity: functions, regulatory mechanisms, and clinical application. Mol Cancer.

